# Hypointense signal lesion on susceptibility-weighted imaging as a potential indicator of vertebral artery dissection in medullary infarction

**DOI:** 10.1038/s41598-024-56134-x

**Published:** 2024-03-04

**Authors:** Euihyun Sung, Wonjae Sung, Young-Jun Lee, So Yeong Jeong, Soo Jeong, Hyun Young Kim, Hyuk Sung Kwon, Seong-Ho Koh, Young Seo Kim

**Affiliations:** 1 Department of Neurology, College of Medicine, Hanyang University, 222 Wangsimniro, Seongdong-gu, Seoul, 04763 Republic of Korea; 2 Department of Radiology, College of Medicine, Hanyang University, Seoul, Republic of Korea; 3 Department of Radiology, Seoul National University Bundang Hospital, Seoul National University College of Medicine, Seongnam, Republic of Korea

**Keywords:** Vertebral artery, Dissection, Susceptibility-weighted imaging, Magnetic resonance imaging, Neuroscience, Neurology

## Abstract

Vertebral artery dissection (VAD) is often associated with medullary infarction; however, an underlying cause may be underestimated. This study aimed to assess the diagnostic potential of hypointense signal lesions along the arterial pathways using susceptibility-weighted imaging (SWI) as a feasible indicator of VAD in medullary infarction. A retrospective analysis was conducted using clinical data, brain magnetic resonance imaging, and angiography records of 79 patients diagnosed with medullary infarction between January 2014 and December 2021. Patients were categorized into an angiography-confirmed dissection group and a non-dissection group based on imaging findings. A new possible dissection group was identified using SWI, including cases with hypointense signals along the arteries without calcification or cardioembolism. We compared the clinical characteristics of the two groups before and after the addition of the hypointense signal as a marker of VAD. The angiography-confirmed dissection group included 12 patients (15%). Among patients lacking angiographic VAD evidence, 14 subjects displayed hypointense signals on SWI: nine patients along the vertebral artery and five subjects at the posterior inferior cerebellar artery without calcification or cardioembolism. The newly classified dissection group was younger, had a lower prevalence of diabetes mellitus and stroke history, and revealed increased headaches compared to the non-dissection group. Hypointense signal detection on SWI in medullary infarctions shows promise as a diagnostic indicator for VAD. Suspicion of VAD is needed when the hypointense signal on SWI is noted, and considering different treatment strategies with angiographic follow-up will be helpful.

## Introduction

Medullary infarctions are uncommon conditions caused by occlusions of the anterior spinal artery, posterior spinal artery, and posterior inferior cerebellar artery (PICA), which originate from the vertebral artery (VA)^1^. The unique blood supply of the medulla has led to investigations into various mechanisms of ischemic stroke, with large artery atherosclerosis emerging as the most common mechanism for both medial medullary infarction (MMI) and lateral medullary infarction (LMI)^2–7^. However, MMI and LMI exhibit distinct etiologies, with small vessel disease predominantly linked to MMI and vertebral artery dissection (VAD) to LMI^4,7,8^. Understanding these differences is crucial, as they lead to varied risk factor prevalence, with conventional risk factors such as diabetes and advanced age being associated with MMI^9^.

Among the causes of medullary infarction, VAD is a significant concern, particularly affecting young ischemic stroke patients^10^. Timely detection of VAD in young patients is essential, as it allows for complete recovery without life-long antithrombotic treatments. Unfortunately, VADs are often underestimated because of the common occurrence of arterial occlusion, making it challenging to establish dissection even with extensive and invasive evaluations. Recently, the identification of hypointense signal lesions in the VA on susceptibility-weighted imaging (SWI) was proposed as an indicator of intramural hematoma, a specific finding of VAD in the absence of calcification and cardioembolic sources^11,12^.

This study aims to evaluate the diagnostic utility of hypointense signal lesions on SWI for diagnosing medullary infarction associated with VAD. We compared clinical characteristics before and after classification of VAD based on the presence of hypointense signal lesions on SWI.

## Results

A total of 79 patients were enrolled in this study, with a mean age (SD) of 60.8 years (13.8) and predominantly male representation (78%). Among these patients, 54 (68%) were diagnosed with LMI, whereas the remaining patients presented with MMI. Routine MRI/MRA screening revealed the presence of VAD in seven patients, with additional confirmation provided by DSA in five patients (15%). Notably, confirmed cases were restricted to the LMI group. Those angiographies showed two intimal flaps, four double lumens, four dissecting aneurysms and nine luminal dilatations plus stenosis. Among the 67 patients who lacked angiographic evidence of dissection, 25 had normal VA, 16 patients exhibited VA stenosis, and 26 had complete occlusion. Notably, 34 patients exhibited a hypointense signal at the VA on SWI, including 11 patients with confirmed angiographic dissection.

**Figure 1:**
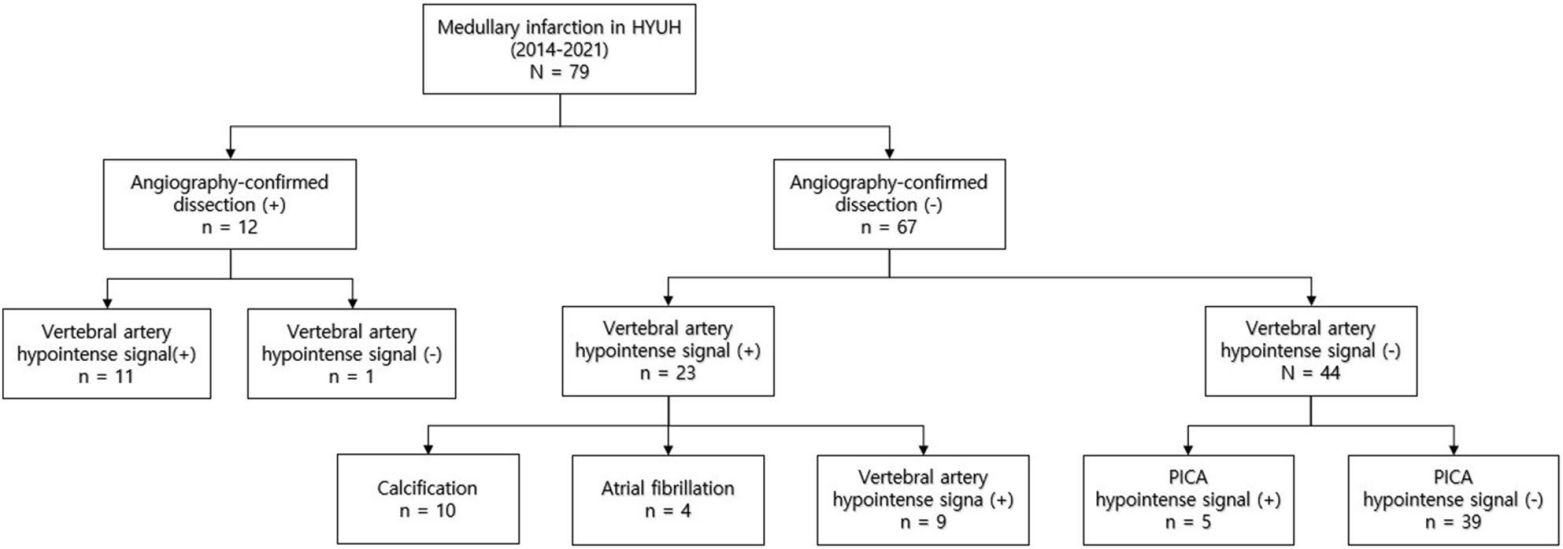
Study flow of the patients.

**Figure 2:**
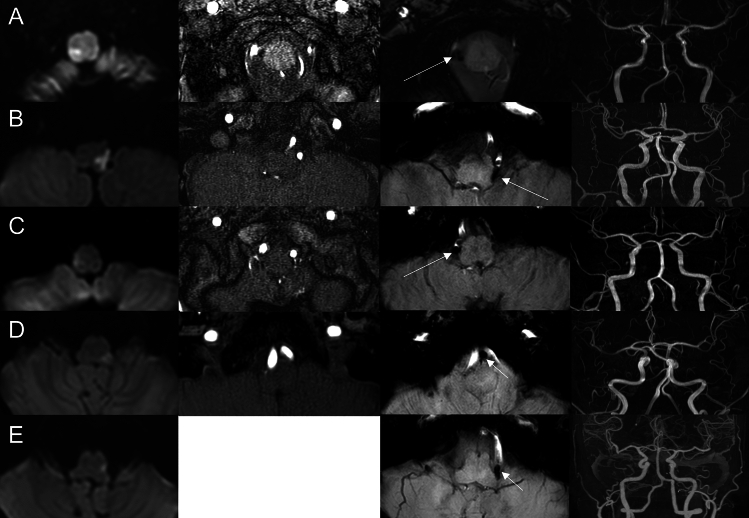
Visualization of diffusion-weighted images, time-of-flight imaging, susceptibility-weighted images (SWI), and magnetic resonance angiography (MRA) in potential posterior inferior cerebellar artery (PICA) dissection patients. The patients presented relatively normal angiographic findings on MRA, but hypointense signal on SWI along the PICA was observed.

Further analysis of patients with hypointense signals but without angiographic evidence of VAD revealed additional factors contributing to this imaging finding. Among the 23 patients in this subgroup, VA calcification was observed in ten subjects, while atrial fibrillation was detected in four patients. Interestingly, nine patients exhibited hypointense signals without these associated findings (Fig. 1). Subgroup analysis of these patients indicated that three individuals had stenotic vertebral arteries and six had occluded vertebral arteries. In contrast, 44 patients did not show hypointense signals in the VA. However, five patients among them presented with a hypointense signal, specifically at the PICA, without any evidence of cardioembolic sources (Fig. 2). When dissection was defined only by the angiography, area under the receiver operating characteristic (ROC) curve (AUC) was 0.854 (Sensitivity = 91.7%, Specificity = 79.1%, Supplementary Fig. 1). Incorporating hypointense signal as a diagnostic marker for VAD resulted in an AUC of 0.981 (Sensitivity = 96.2%, Specificity = 100.0%, Supplementary Fig. 1).

**Figure 3:**
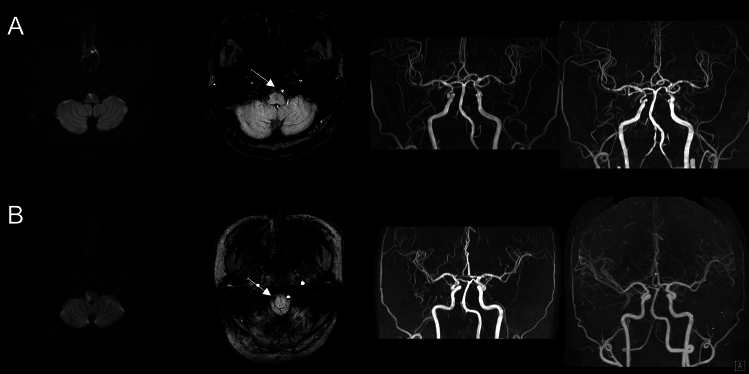
Representative images of patients with medial medullary infarction (**A**) and lateral medullary infarction (**B**) displaying hypointense signal on SWI, without definitive evidence of dissection on angiography. Both patients showed partial recanalization of occluded vertebral artery 1 year after the symptom onset.

Long-term follow-up angiography was performed on a subset of patients approximately 1 year after the initial ischemic stroke event. Notably, two patients with initially occluded vertebral arteries and corresponding hypointense signals demonstrated partial recanalization of the affected arteries after 1 year, suggesting VAD as the underlying etiology (Fig. 3).

A comparative analysis was conducted to assess the distinguishing features of patients with and without angiographically confirmed VAD and those without. Patients with confirmed dissection were younger, less likely to have diabetes mellitus, and reported a higher incidence of headache. However, when dissection was defined by incorporating the presence of a hypointense signal on SWI and excluding calcification and cardioembolic sources, differences in age, diabetes mellitus, and headache became more pronounced. Additionally, this novel classification demonstrated a significantly lower prevalence of stroke history compared with conventional angiographic confirmation (Table 1).

**Table 1:** Characteristics of patients with medullary infarction according to the presence of vertebral artery dissection.

	Dissection by angiography (n = 79)			Dissection by angiography and SWI (n = 79)		
	Dissection (+) (n = 12)	Dissection (−) (n = 67)	*P* value	Dissection (+) (n = 26)	Dissection (−) (n = 53)	*P* value
Demographics						
Age, years (SD)	51.5 ± 12.1	62.4 ± 13.5	0.011	50.8 ± 11.9	65.6 ± 12.1	< 0.001
Sex, male (%)	9 (75.0)	53 (79.1)	0.714	18 (69.2)	44 (83.0)	0.161
Clinical findings						
History of TIA, n (%)	0 (0)	2 (3.0)	1.000	0 (0)	2 (3.8)	1.000
History of stroke, n (%)	0 (0)	9 (13.4)	0.341	0 (0)	9 (17.0)	0.026
Coronary artery disease, n (%)	0 (0)	3 (4.5)	1.000	0 (0)	3 (5.7)	0.547
Atrial fibrillation, n (%)	1 (8.3)	6 (9.0)	1.000	1 (3.8)	6 (11.3)	0.416
Hypertension, n (%)	7 (58.3)	47 (70.1)	0.504	17 (65.4)	37 (69.8)	0.691
Diabetes mellitus, n (%)	0 (0)	29 (43.3)	0.003	4 (15.4)	25 (47.2)	0.006
Dyslipidemia, n (%)	8 (66.7)	51 (76.1)	0.487	19 (73.1)	40 (75.5)	0.818
Current smoker, n (%)	3 (25.0)	22 (32.8)	0.743	7 (26.9)	18 (34.0)	0.527
Headache, n (%)	8 (66.7)	14 (33.3)	0.003	13 (50.0)	9 (17.0)	0.003
Initial NIHSS, median (IQR)	2.5 (1–3.25)	2 (1.5–4)	0.554	2 (1–3)	2 (2–4)	0.289
Discharge NIHSS, median (IQR)	1 (1–2)	1 (0–3)	0.644	1 (0.5–2)	1 (0–3)	0.118
Discharge mRS, median (IQR)	1 (1–2)	1 (1–2.5)	0.470	1 (1–2)	2 (1–3)	0.067
Radiologic findings						
Location of infarction						
Lateral medulla, n (%)	12 (100)	42 (62.6)		25 (96.2)	29 (54.7)	
Medial medulla, n (%)	0	25 (37.3)		1 (3.8)	24 (45.3)	
Vertebral artery by angiography						
Normal, n (%)	0 (0)	25 (37.3)		5 (19.2)	20 (37.7)	
Dissection, n (%)	12 (100)	0 (0)		12 (46.2)	0 (0)	
Stenosis, n (%)	0 (0)	16 (23.9)		3 (11.5)	13 (24.5)	
Occlusion, n (%)	0 (0)	26 (38.8)		6 (23.1)	20 (37.7)	

## Discussion

In this study, we found that hypointense signals on SWI without calcification or cardioembolic sources may be useful indicators for the diagnosis of VAD. The inclusion of this SWI marker allowed for a more prominent differentiation of risk factors and symptom severity between patients with and without VADs. Notably, the majority of patients with LMI and confirmed VAD exhibited a hypointense signal. Interestingly, some patients without angiographic evidence of VAD also presented with a hypointense signal, suggesting its possible role as an indicator of intramural hematoma.

VAD is a condition in which an intimal tear leads to the formation of an intramural hematoma, resulting in a false lumen within the layers of the tunica media. This condition can cause VA steno-occlusion or aneurysmal dilatation, leading to both ischemic and hemorrhagic stroke^13^. Confirming VAD typically involves catheter angiography and high-resolution MRI, which reveal characteristic features such as intimal flap, double lumen, narrowing, occlusion, dilatation, and pseudoaneurysms^3^. However, when routine angiographic findings appear normal or complete occlusion, it leads to dismiss the possibility of arterial dissection. SWI has been used to detect intracerebral hemorrhage, hemorrhagic transformation, vascular malformations, microbleeds and oxygenation status of venous blood and arterial thrombus^14^. In conjunction with phase maps and non-contrast brain computed tomography, SWI was used to define intramural hematoma in VAD, excluding calcification^11,15,16^. In this study, we found an additional nine patients among 79 patients with medullary infarction who showed hypointense signals in the VA without calcification or cardioembolic sources. These patients exhibited VA stenosis or obstruction without evidence of VAD on routine brain MRI or MRA. In addition, we observed five patients with hypointense signals at the PICA among those with normal VAs, which may be associated with PICA dissection. These observations are in line with those of previous case series studies, suggesting that PICA hypointense signals on SWI are indicators of PICA dissection^17^. Although, some patients may associated with embolic stroke of undetermined sources, we believe that those hypointense signals may be associated with dissection in many cases where identifiable embolic sources are absent.

Medullary infarction represents a small proportion of all cerebral infarctions, primarily due to abundant collateral circulation. The classification of medullary infarction into MMI and LMI is based on the location involved; distinct risk factors and stroke mechanisms have been proposed for these subtypes. Specifically, age, diabetes mellitus, and atherosclerosis were identified as independent risk factors for MMI relative to LMI^2,9^. Since risk factors are different between MMI and LMI, different stroke mechanism has been also suggested in many studies. Generally, large artery atherosclerosis has been the most common mechanism of both MMI and LMI in a few studies^4–7,9^. However, the second most common mechanism in these two diseases has been demonstrated differently. Small vessel disease was shown to be more associated with MMI, and VAD has been shown to be associated with LMI^2,4,7^. In one study, the second most common mechanism for LMI was small vessel disease rather than arterial dissection, but this study had rather tight arterial dissection criteria which needs angiographic findings with concurrent neck or occipital pain^9^. In this study, we identified that the most common mechanism of both MMI and LMI was large artery atherosclerosis. The second most common mechanism for LMI was VAD (22%, 12 of 54 patients), which was in line with previous reports^4,7^. After adding a VA hypointense signal on SWI as an arterial dissection, the proportion of VAD increased up to 37% (20 of 54 patients). Moreover, five patients showed hypointense signals on the PICA, and if we added those patients as dissection, the rate of dissection on LMI was 46% (25 of 54 patients). Although 60% (15 of 25 patients) showed a steno-occlusive VA in patients with MMI, only one patient showed a hypointense signal on SWI without calcification.

The detection of VAD in medullary infarction is clinically important because of its different outcomes and treatment strategies. Patients with VAD have relatively favorable outcomes compared to those with intracranial atherosclerosis-related ischemic stroke^12,18^. In addition, patients with VAD-related medullary infarction do not need life-long antithrombotic treatment, and guidelines recommend 3 months of antiplatelet or anticoagulation treatment^19^. Therefore, discriminating VAD from other stroke mechanisms could be important for patients, especially arterial dissection-related ischemic stroke, which occurs in relatively young patients. To the best of our knowledge, the SWI hypointense signal is not a perfect diagnostic marker for arterial dissection; it can serve as a valuable tool to raise suspicion in patients presenting with this imaging finding.

This study had certain limitations. This was a retrospective observational study conducted at a single center with a relatively small sample size. However, our inclusion criteria focused solely on patients with pure medullary infarction without concomitant extramedullary infarction, thus providing unique insights into the relationship between medullary infarction and VAD. Further investigations with larger sample sizes are warranted to elucidate the characteristics of patients with medullary infarctions and hypointense signals on SWI. Additionally, we did not perform diagnostic angiography in all patients to detect VAD as this was not a routine procedure when patients exhibited steno-occlusive vertebral arteries without evidence of dissection. Moreover, the appearance of a hypointense signal on SWI may be time-sensitive and related to the onset of dissection^20^. While our inclusion criteria limited the patient population to those within 7 days of ischemic stroke onset, the time difference from dissection onset to MRI might influence the presence of the hypointense signal. Finally, the study was conducted exclusively on the Korean population, limiting the generalizability of the results to other ethnic groups.

In conclusion, a hypointense signal on SWI may serve as a potential marker for VAD in patients with medullary infarction. These patients exhibited less severe strokes, favorable outcomes, and fewer conventional risk factors. This finding suggests that there may be a higher proportion of patients with arterial dissection in the LMI group than previously reported. As these patients may not require lifelong antithrombotic therapy, follow-up angiography to detect improvement in arterial dissection could be helpful in diagnosing VAD. However, further multicenter studies with larger cohorts are warranted to validate the utility of the SWI hypointense signal as a diagnostic tool for VAD in different populations and to determine its clinical implications for treatment strategies.

## Methods

### Study population

This retrospective study utilized a prospectively and consecutively enrolled hospital-based registry to identify patients who visited Hanyang University Hospital due to acute ischemic stroke within 7 days of initial symptoms between January 2014 and December 2021. Among 2300 patients, a total of 79 patients (3.4%) presenting with ischemic stroke localized to the medulla were identified through diffusion-weighted imaging (DWI). This study was approved by the Institutional Review Board of Hanyang University Hospital (HYUH 2023-04-055). The requirement for informed patient consent was waived due to the retrospective use of data. All the methods and procedures carried out in this study were in accordance with relevant guidelines and regulation.

### Demographic characteristics and risk factors

Demographic data (age and sex) and risk factors (hypertension, diabetes, dyslipidemia, smoking, and history of previous stroke or transient ischemic attack) were collected upon admission. Hypertension was defined as the previous use of antihypertensive medication, systolic BP > 140 mmHg, or diastolic BP > 90 mmHg at discharge BP 1 week after symptom onset. Diabetes was defined as previous use of antidiabetic medication, fasting blood glucose ≥ 126 mg/dl, or hemoglobin A1C ≥ 6.5%, and dyslipidemia as previous use of lipid-lowering agents or low-density lipoprotein levels ≥ 100 mg/dl. The subjects were classified as current smokers or non-smokers. To investigate the embolic source, all patients underwent transthoracic echocardiography and 24-h holter monitoring. Upon admission, stroke severity was assessed using the National Institutes of Health Stroke Scale (NIHSS) at admission.

### Image acquisition and analysis

All patients underwent brain computed tomography, magnetic resonance imaging (MRI) and magnetic resonance angiography (MRA) within 48 h of admission. All MRI scans were obtained using a 3-Telsa MR machine (Achieva, Philips, Best, Netherlands) with an eight-channel sensitivity-encoding coil. The standardized protocol consisted of axial DWI, axial fluid attenuated inversion recovery (FLAIR), SWI, T1 weighted image with contrast enhancement (T1CE) and MRA. Conventional DWI (TR/TE = 4000/73 ms, slice thickness/gap = 5/2 mm, matrix size = 128 × 128, FOV = 240 × 240 mm), FLAIR (TR/TE/TI = 11,000/125/2800 ms, slice thickness/gap = 5/2 mm; matrix size = 352 × 189, FOV = 230 × 183 mm), SWI (TR/TE = 31/7 ms, slice thickness/gap = 5/2 mm, matrix size = 368 × 367, FOV = 24 × 24 cm), T1CE (TR/TE = 7/4, slice thickness/gap = 5/2 mm, matrix size = 304 × 303, FOV = 24 × 24 mm) were obtained by using the appropriate parameters. MRA was performed using the 3D time-of-flight technique with a 3D spoiled gradient recalled-echo pulse sequence with the following parameters: TR/TE/flip angle = 25/3.45 ms/20°, matrix = 512 × 208, FOV = 20 cm, section thickness = 0.8 mm and effective voxel size = 0.39 mm × 0.96 mm × 0.8 mm. Source image data were reformatted using maximal intensity projections in both vertical and horizontal directions. Digital subtraction angiography (DSA) was performed to diagnose arterial dissection when additional treatment was considered necessary.

**Figure 4:**
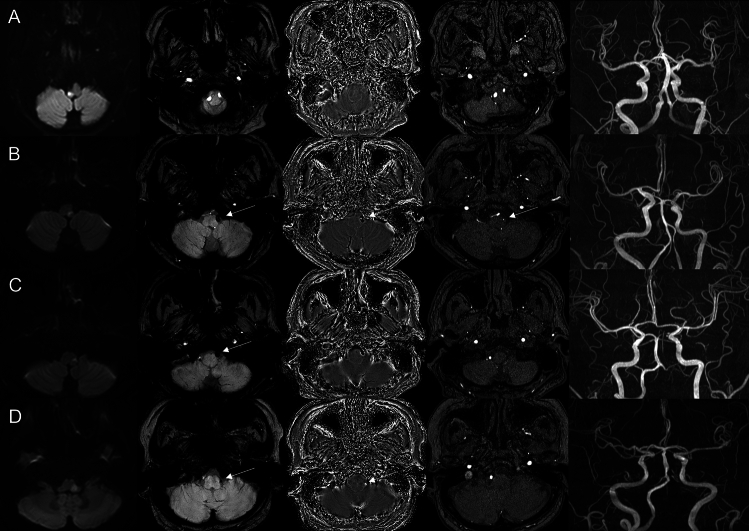
Illustrative examples of different cases of lateral medullary infarction, showcasing various imaging modalities. The images include diffusion-weighted imaging (DWI), susceptibility-weighted imaging (SWI), SWI phase maps, time-of-flight magnetic resonance angiography (TOF-MRA), and MRA. (**A**) Right lateral medullary infarction without hypointense signal on SWI and normal angiography on MRA. (**B**) Left lateral medullary infarction with hypointense signal on SWI, displaying possible intramural hematoma and intimal flap on TOF-MRA, suggestive of dissection. (**C**) Left lateral medullary infarction with hypointense signal on SWI and occlusion on MRA. (**D**) Left lateral medullary infarction with hypointense signal on SWI, accompanied by a hyperintense signal on the SWI phase map, indicating vessel calcification. MRA demonstrates occlusion of the left vertebral artery.

Patients were classified into the angiography-proven dissection group when typical radiologic characteristics (intimal flap, double lumen, dissecting aneurysm or luminal dilation plus stenosis) in at least one confirmatory angiographic examination including MRA and DSA^3^. Patients were classified into the possible dissection group when a hypointense signal in the VA was present on SWI. A hypointense signal was defined when it was along the relevant vessel, either the distal VA or PICA on SWI. Hypointense signals with computed tomography (CT) and SWI phase-map-proven calcification and probable embolisms from cardiac sources, such as atrial fibrillation, valvular heart disease and akinetic ventricular segments were excluded after transthoracic echocardiography and 24 h holter monitoring. Radiologic findings were assessed by independently by two experienced neurologists (ES and YSK). Discrepancies between the two readers were resolved by consensus with neuroradiologist (YJL). Figure 4 shows various patient examples based on DWI, SWI, SWI phase maps, and time-of-flight magnetic resonance angiography.

### Statistical analysis

Continuous variables are presented as means ± standard deviation and were analyzed using Student’s *t*-test. Non-parametric variables are presented as median (interquartile range) and were analyzed using Mann–Whitney U Test. Categorical variables are presented as numbers (%) and analyzed by χ^2^ tests. The Fisher's exact test was used when the number of cells was small. All statistical analyses were performed using the SPSS for the Social Sciences (version 28.0). *P* values < 0.05 were considered as significant difference.

### Supplementary Information

Supplementary Figure 1.

## Data Availability

The datasets used and/or analyzed during the current study are available from the corresponding author on reasonable request.
