# Supplementary material for: Hypointense signal lesion on susceptibility-weighted imaging as a potential indicator of vertebral artery dissection in medullary infarction
Source: Sci Rep. 2024 Mar 4;14:5258. doi: 10.1038/s41598-024-56134-x (PMC10912406; doi:10.1038/s41598-024-56134-x)
Supplement: Supplementary file 1 — Supplementary Figure 1. [file 41598_2024_56134_MOESM1_ESM.docx]

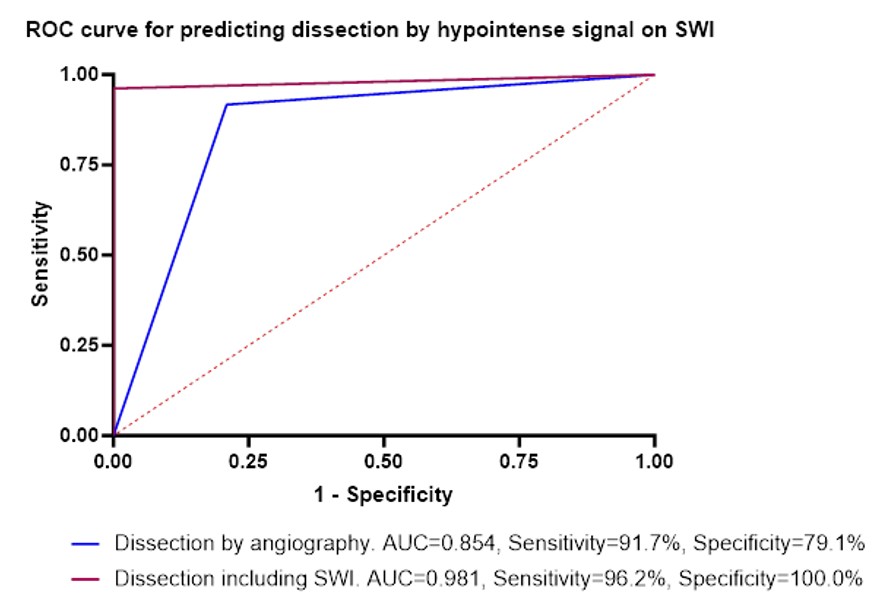


Supplementary Figure 1. ROC curve for predicting dissection by hypointense signal on SWI.

When dissection was defined only by the angiography (blue line), AUC was 0.854 (Sensitivity=91.7%, Specificity=79.1%). Incorporating hypointense signal as a diagnostic marker for dissection (red line), AUC was 0.981 (Sensitivity=96.2%, Specificity=100.0%). ROC, Receiver operating characteristic; SWI, susceptibility weighted imaging; AUC, area under curve
